# Using Generative AI to Appraise the Quality of Medical Education Research Studies: Agreement Between AI‐Generated and Human MERSQI Scores

**DOI:** 10.1002/aet2.70189

**Published:** 2026-05-14

**Authors:** Mohammad Aldalou, William S. Brooks, Emily Rush, Bernard Landry‐Wegener, Corey Bills, David A. Cook, John Swope, Adam B. Wilson

**Affiliations:** ^1^ Department of Medical Education, Marnix E. Heersink School of Medicine University of Alabama at Birmingham Birmingham Alabama USA; ^2^ Office of Institutional Effectiveness, Academic Affairs Rush University Chicago Illinois USA; ^3^ Department of Anatomy and Cell Biology, Rush Medical College Rush University Chicago Illinois USA; ^4^ Department of Internal Medicine Johns Hopkins School of Medicine Baltimore Maryland USA; ^5^ Department of Emergency Medicine, Anschutz School of Medicine University of Colorado Aurora Colorado USA; ^6^ Department of General Internal Medicine Mayo Clinic Rochester Minnesota USA; ^7^ Curricu.me Londonderry New Hampshire USA

**Keywords:** AI‐assisted research, artificial intelligence, medical education, MERSQI

## Abstract

**Objectives:**

The increasing volume of medical education research necessitates efficient, reliable, and scalable methods for conducting quality appraisals. The Medical Education Research Study Quality Instrument (MERSQI) is a widely used tool, although its manual scoring process remains resource‐intensive. This study evaluated how well large language models (LLMs) appraise medical education research using the MERSQI tool in comparison with human judges.

**Methods:**

Three LLMs (GPT‐5, Claude Sonnet 4, and Gemini 2.5 Pro) assigned MERSQI domain scores to 1423 medical education research articles. The authors compared AI‐generated scores with human‐generated scores using intraclass correlation coefficients (ICCs) across the six MERSQI domains. They evaluated the agreement between AI‐ and human‐generated MERSQI composite scores using Bland–Altman plots.

**Results:**

Domain‐level ICC values ranged from fair (0.24) to near perfect (0.81), with the lowest agreement observed in the ‘sampling,’ ‘validity evidence,’ and ‘data analysis’ domains. No single LLM consistently outperformed the others across all domains. Composite score agreement with human ratings was substantial and similar across LLMs (ICC range: 0.65–0.69). GPT‐5 produced slightly lower composite scores than humans, while Claude Sonnet 4 and Gemini 2.5 Pro produced higher scores, with Gemini showing the largest deviation. The Bland–Altman plots for Gemini 2.5 Pro suggested proportional bias, indicating its agreement with human scores varied across the range of study quality.

**Conclusions:**

These LLMs demonstrated substantial agreement with human raters for MERSQI composite scores, but domain‐level agreement varied. Systematic differences in scoring patterns highlight the need for human oversight and additional calibrations before integrating LLMs into systematic review appraisal workflows.

## 1 Introduction

Rigorous quality appraisals are an important aspect of systematic reviews, as they help identify low‐quality studies and justify the validity of synthesized conclusions [1, 2]. Quality appraisals systematically examine the methodological quality and trustworthiness of empirical research by assessing its relevance to a specific review question or context and verifying whether a study's design, sampling, data collection, analysis, and reporting are appropriate and transparent [3]. Often conducted in duplicate, quality appraisals of empirical studies are labor‐intensive as they demand considerable human capital, time, and expertise to meticulously evaluate each study against predefined quality criteria [4]. As the volume of research literature expands, and with it the number and size of literature reviews, such demands could hinder the timely reporting of synthesized evidence or dissuade investigators from conducting quality appraisals altogether. These burgeoning constraints, coupled with incidental human discrepancies, errors, and oversights, underscore the need for more efficient, consistent, and feasible methodologies.

Artificial intelligence (AI) has shown promise in automating complex tasks and supporting large‐scale data processing. Over the past decade, researchers have increasingly explored the use of machine learning to automate quality appraisal processes across various domains, including evidence‐based medicine and education. Existing tools to appraise research quality include RobotReviewer for randomized controlled trials [5] and SciScore for life sciences research [6]. Similar AI methods have also been used to predict expert ratings in national research appraisals such as the United Kingdom's Research Excellence Framework [5, 7]. Advancements in both domain‐agnostic and specialized GenAI tools may offer greater rigor for quality appraisals by increasing objectivity and consistency in fields where the volume of research output overwhelms traditional quality appraisal workflows [1]. While early applications are encouraging, the use of GenAI for appraising research quality remains in its infancy and requires further validation [8, 9].

A body of literature in clinical medicine suggests that GenAI has the potential to critically appraise study methods; however, evidence for this specific use case is limited [10, 11, 12]. For example, only a small proportion of software tools for supporting the automation of systematic review processes have been tested for risk of bias assessment, and most were applied to specific study designs, such as randomized controlled trials [10]. Not all tools were compared against human evaluators, and they generally needed further validation using larger datasets across a wider range of topics [10]. In a related review of software systems aimed at enhancing the efficiency of systematic reviews, Affengruber et al. found that RobotReviewer was the only tool evaluated as a critical appraisal system, highlighting a lack of performance data for other critical appraisal systems [11]. These findings underscore a gap in validating newer GenAI tools for appraising research quality beyond clinical trials. This gap is particularly relevant to medical education research, where study designs are often heterogeneous (e.g., qualitative studies, quasi‐experimental studies, and curriculum evaluations) and constituent disciplines may compound variability further. For example, Emergency medicine education includes research on simulation‐based procedural training, point‐of‐care ultrasound curricula, residency assessment, and team‐based clinical decision‐making, often with variable outcome measures and inconsistent reporting standards [13, 14, 15]. Systematic reviews synthesizing this scholarship must navigate this complexity, as outcomes may influence training program designs, accreditation standards, clinical workflows, and the quality of patient care delivered by graduates [16].

This inherent variability makes medical education both a useful test case and a demanding setting for investigating whether newer GenAI systems can apply established appraisal criteria across broad, methodologically diverse literature. The Medical Education Research Study Quality Instrument (MERSQI) provides an established mechanism for assessing the methodological quality of empirical quantitative studies. Prior studies have reported good to excellent interrater reliability for MERSQI scores and have shown associations between higher scores and markers of scholarly quality, including research funding and editorial acceptance decisions [17, 18, 19, 20, 21].

This study examined how well MERSQI scores derived from three commercial LLMs (GPT‐5, Claude Sonnet 4, and Gemini 2.5 Pro) agreed with human‐generated scores at the composite and individual domain score levels and whether systematic scoring differences existed between AI and human raters. Understanding GenAI's ability to apply the MERSQI tool may support its use as a second coder or to automate certain research tasks under human supervision. This, in turn, may enhance the consistency, scalability, and routine implementation of quality appraisals in medical education research syntheses.

## 2 Methods

### 2.1 Medical Education Research Study Quality Instrument

The Medical Education Research Study Quality Instrument (MERSQI) is a tool for appraising the methodological quality of medical education research studies. It evaluates six domains: study design, sampling, type of data, validity evidence, data analysis, and outcomes. Each domain consists of specific criteria, with scores ranging from 0 to 3 or 1 to 3, depending on the category. A composite MERSQI score is derived by summing the scores across the domains, with a maximum score of 18. In practice, it is recommended and more meaningful to report and interpret MERSQI domain scores over composite scores to better gauge a study's methodological strengths and limitations. Higher domain or composite scores indicate stronger methodological quality in medical education research [17]. For terms and technologies referenced throughout this manuscript, see Table 1.

**TABLE 1:** Key terms and technologies.

Term	Description
AI	Artificial intelligence; computational systems designed to perform tasks that typically require human cognition, such as pattern recognition, prediction, classification, reasoning, or language generation.
GenAI	Generative artificial intelligence; AI systems that generate new content, such as text, data, code, images, or summaries, in response to user prompts.
LLM	Large language model; a type of generative AI trained on large text corpora to process, interpret, and generate human language.
GPT‐5	A large language model developed by OpenAI and evaluated in this study as one of the commercial LLMs used to generate MERSQI scores.
Claude Sonnet 4	A large language model developed by Anthropic and evaluated in this study as one of the commercial LLMs used to generate MERSQI scores.
Gemini 2.5 Pro	A large language model developed by Google and evaluated in this study as one of the commercial LLMs used to generate MERSQI scores.
MERSQI	Medical Education Research Study Quality Instrument; a six‐domain instrument used to appraise the methodological quality of empirical quantitative medical education research.
API	Application programming interface; a software interface that enables programmatic interaction with digital systems, including AI platforms.
JSON	JavaScript Object Notation; a structured, machine‐readable format commonly used to organize and return data from software systems or AI outputs.

### 2.2 Data Collection and Eligibility Criteria

This study compares the accuracy of AI‐generated MERSQI scores to human‐generated scores from a convenience sample of previously published systematic reviews. The dataset collected MERSQI scores from original published studies, or if data were unpublished, directly from the original authors. Both approaches, detailed below, were undertaken in parallel until the target sample size of at least 1000 MERSQI‐scored studies was achieved. According to a power analysis (conducted in R [22] using the “ICC.Sample.Size” package), to detect an intraclass correlation coefficient (ICC) of 0.20 (slight agreement) versus 0.30 (fair agreement) at a power of 0.90, a sample size of at least 923 MERSQI‐scored studies was required (*k* = 2 ratings, 2‐sided, alpha = 0.05).

#### 2.2.1 Approach 1—Unpublished Datasets

A search was conducted through MedEdMentor using the keyword “MERSQI” to identify review articles where authors applied the full MERSQI tool to appraise at least 20 education‐focused studies published in clinical or medical education journals. For reviews that did not publish the individual study‐level MERSQI scores, we contacted the corresponding authors, asking them to provide the study‐level scores.

#### 2.2.2 Approach 2—Published Datasets

We identified additional datasets through PubMed using the keyword “MERSQI” to find reviews that published their study‐level MERSQI data (e.g., in the main text or as supplemental appendices). Starting with the most recently published reviews, we examined each published study and selected for inclusion those with publicly available data until the target sample size was achieved.

#### 2.2.3 Eligibility Criteria

We included studies that addressed any medical education topic. No date or year restrictions were applied. Works in related healthcare disciplines/journals (e.g., dental education, nursing) were excluded. We included only reviews with study‐level human‐generated MERSQI scores across all six domains that used original (unmodified) MERSQI scoring definitions. If an original research study was included in more than one review, scores from the first published review were used. The present study did not require ethical approval as it did not involve human subjects.

### 2.3 LLMs and Prompting Procedure

Three commercial LLMs were selected for evaluation: GPT‐5 (OpenAI, San Francisco, CA; openai.com), Claude Sonnet 4 (Anthropic, San Francisco, CA; anthropic.com), and Gemini 2.5 Pro (Google DeepMind, Mountain View, CA; deepmind.google.com). These represented the most advanced available model from each of the three most widely used commercial AI platforms at the time of this study. Each was prompted to appraise the methodological quality of medical education research studies, with human‐generated MERSQI scores serving as the reference standard.

We obtained the full text PDF of each included study and converted them to machine‐readable text using PyMuPDF, pdfplumber, or OCR (pytesseract), in that order, until acceptable text was returned. We then used Jupyter Notebook and application programming interfaces (APIs) to upload each study text to each LLM. For GPT‐5, the reasoning effort was set to “minimal,” the verbosity to “low,” and all other settings were left at their default values. For Claude Sonnet 4 and Gemini 2.5 Pro, the temperature was set to 0.5 (i.e., moderately low randomness) to ensure more focused and consistent outputs. Other configurations were left at their default values.

We crafted a prompt that requested structured JavaScript Object Notation (JSON) outputs, including a one‐sentence article summary, MERSQI domain scores, justifications, and other metadata. The full prompting protocol and JSON schema are available as supplemental material accompanying the online article (Data S1). We used the same zero‐shot prompt for all three LLMs to establish their baseline performance without prompt optimization or refinements. Results were aggregated into a pandas DataFrame, where domain‐level scores were summed to obtain a total composite score and then exported to timestamped Excel files for further processing. This automated approach enabled efficient, reproducible, and scalable quality appraisal of a large corpus of medical education research articles.

### 2.4 Statistical Analysis

The primary outcome was the degree of agreement between each LLM and human‐generated MERSQI scores across composite and individual domain score levels. The secondary outcome was the presence and magnitude of systematic scoring differences between AI and human raters. All statistical analyses were conducted in SPSS (version 29, IBM Corp., Armonk, NY).

Domain‐ and composite‐level agreement between each LLM and human raters was assessed using a two‐way random effects intraclass correlation coefficient (ICC [1, 2]) for absolute agreement. Because no externally validated “true” MERSQI score exists independently of human judgment, human ratings served as the reference standard, and AI‐human agreement is interpreted throughout as a proxy measure of AI scoring accuracy. Agreement was interpreted using criteria by Landis and Koch with fair (0.21–0.40), moderate (0.41–0.60), substantial (0.61–0.80), and almost perfect (> 0.80) agreement [23].

Systematic differences in mean MERSQI scores between each LLM and human raters were examined using repeated measures ANOVA. Effect sizes were measured using partial eta‐squared (*η* ^2^) and were interpreted as small (0.01–0.05), medium (0.06–0.13), or large (≥ 0.14) effects [24]. Alpha was set at 0.05, and outcomes are reported as means (*μ*) and standard deviations (SD). Bland–Altman plots were used to visually assess the agreement between AI‐generated and human MERSQI composite scores. A linear regression of the score differences on the means tested for the presence of proportional bias (i.e., whether the measurement differences varied with score averages) [25]. A significant result (*p* < 0.050) for proportional bias indicates that the methods being compared do not agree equally across the range of values.

## 3 Results

### 3.1 Data Collection Outcomes and Summary of Study Features

#### 3.1.1 Data From Unpublished Datasets

The MedEdMentor search yielded 25 review articles that met the inclusion criteria. The corresponding authors of these 25 articles were invited to share their article‐level MERSQI data underlying their published reviews. Three corresponding authors responded with their unpublished datasets, contributing 1334 individually MERSQI‐scored articles in total.

#### 3.1.2 Data From Published Datasets

The PubMed search returned 174 unique records, which underwent screenings and full‐text reviews in the sequence of the returned records. Of these, three published reviews, comprising a total of 89 MERSQI‐scored studies, were added to the dataset. Further efforts to acquire additional published datasets were discontinued upon receiving one large dataset of 1000+ MERSQI‐scored studies from one corresponding author [26, 27]. The final dataset for analysis consisted of 1423 MERSQI‐scored articles, compiled by combining the unpublished and published datasets.

The MERSQI‐scored studies were published between 1969 and 2021. Human MERSQI scores were generated by research teams from contributing systematic reviews. Scores were derived either by a single individual (3.0%; 43/1423) or through consensus after blinded independent reviews by two reviewers (97.0%; 1380/1423). However, detailed characteristics of individual raters, including their level of formal MERSQI training and years of experience, were not reported across the contributing reviews and could not be extracted or presented.

The sources of the MERSQI‐appraised studies and score derivation information for all 1423 articles are presented as supplemental material accompanying the online article (Table S1).

### 3.2 Domain Score Agreement and Analysis

**FIGURE 1:**
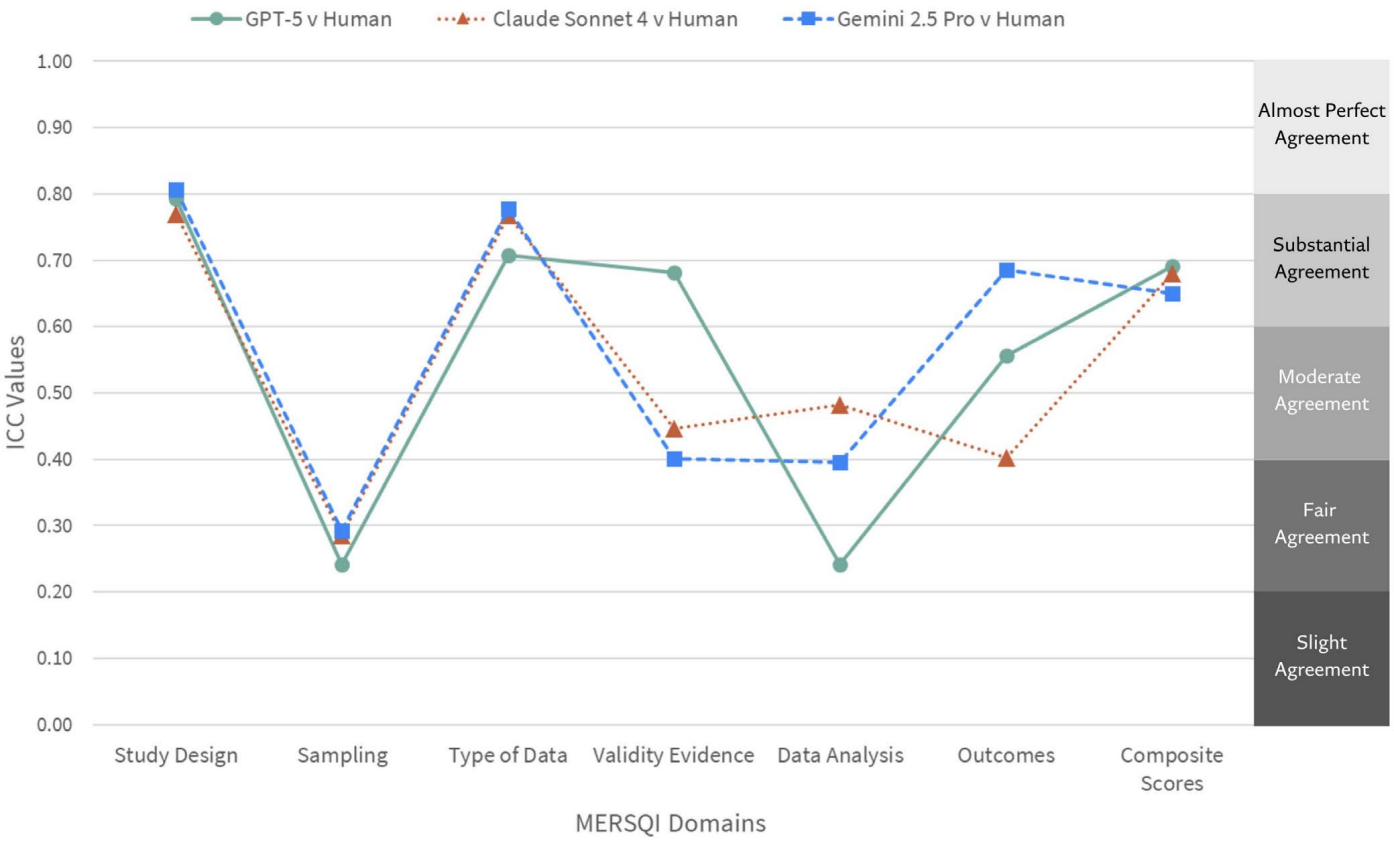
Intraclass correlation coefficient (ICC) values comparing agreement between the large language models (LLM) and human raters across the Medical Education Research Study Quality Instrument (MERSQI) composite and domain scores. Lines indicate GPT‐5 (green circles, solid), Claude Sonnet 4 (orange triangels, dotted), and Gemini 2.5 Pro (blue squares, dashed). Higher ICC values reflect stronger agreement between the LLM model and human scoring.

**FIGURE 2:**
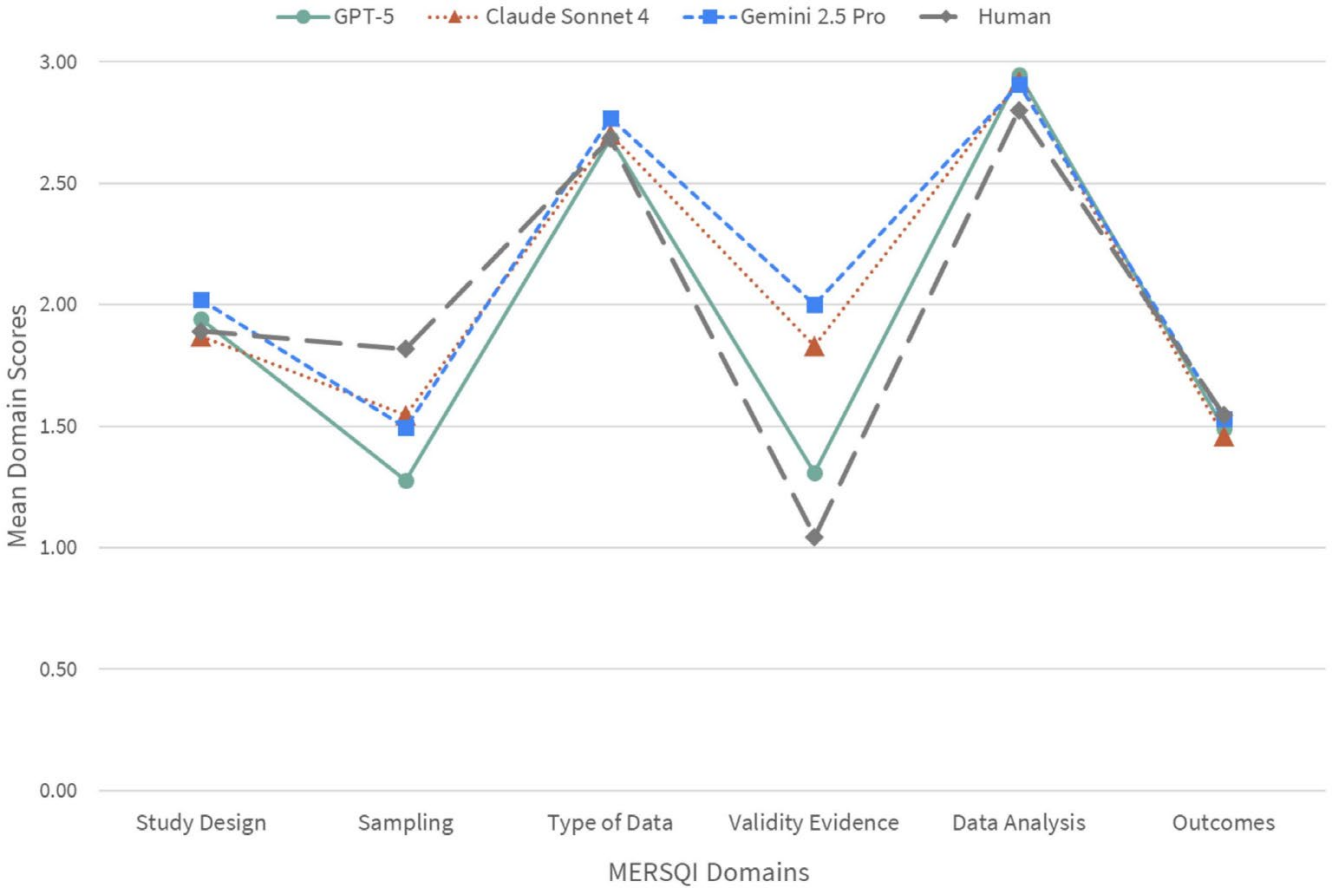
Mean Medical Education Research Study Quality Instrument (MERSQI) domain scores for the large language models (LLMs) and human raters. Lines represent GPT‐5 (green circles, solid), Claude Sonnet 4 (orange triangles, dotted), Gemini 2.5 Pro (blues squares, dashed), and human raters (gray diamonds, dashed).

Inter‐rater agreement between the LLMs and human raters was conducted across 1423 articles for each of the six MERSQI domains and composite scores. Domain‐level ICC values ranged from fair (ICC = 0.24; 95% CI = −0.02 to 0.44) to near‐perfect agreement (ICC = 0.81; 95% CI = 0.76 to 0.84; Figure 1). No single LLM outperformed the others across all domains (Figure 1). ICC values were generally the lowest for the sampling, validity evidence, and data analysis domains. Figure 2 presents AI versus human mean domain scores. On average, humans tended to assign higher scores to the sampling domain (*p* < 0.001; *η* ^2^ ≥ 0.14, large effect) and lower scores to the validity evidence domain (*p* < 0.001; *η* ^2^ ≥ 0.61, large effect) compared to the LLMs (Figure 2). Effect sizes for differences between human and LLM scores among the remaining four domains were medium to small (*η* ^2^ ≤ 0.08).

### 3.3 Composite Score Agreement and Analysis

**FIGURE 3:**
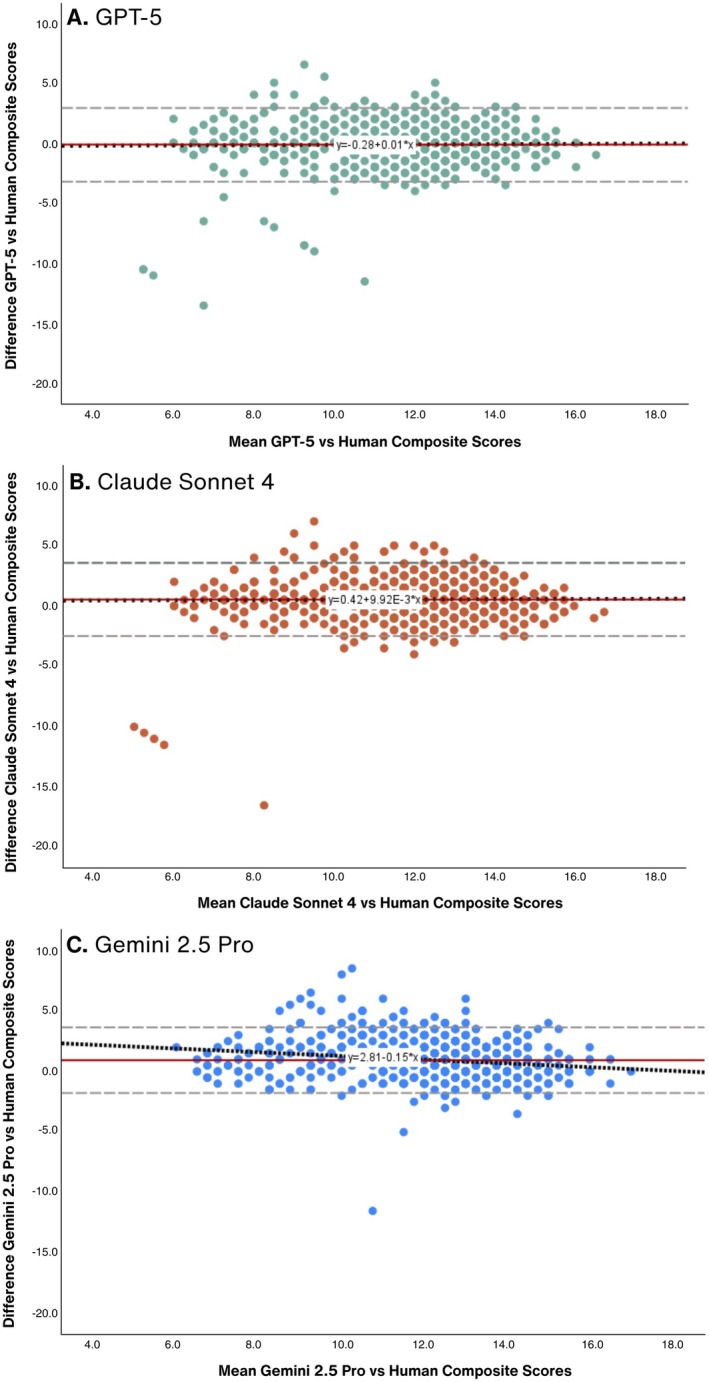
(A–C) Bland–Altman plots comparing composite Medical Education Research Study Quality Instrument (MERSQI) scores assigned by each large language model (LLM) and human raters. The *x*‐axis shows the mean of LLMs and human scores. The *y*‐axis shows the score difference (LLM model minus human). The solid red line represents the mean difference, the gray dashed lines indicate the 95% limits of agreement, and the black dotted line represents a trend line. A downward‐sloping trend line indicates that the LLM assigned higher scores than human raters at lower mean MERSQI values and lower scores at higher mean values, consistent with proportional bias. (A) GPT‐5 versus human raters: mean difference = −0.14; proportional bias test *p* = 0.566. (B) Claude Sonnet 4 versus human raters: mean difference = +0.53; proportional bias test *p* = 0.653. (C). Gemini 2.5 Pro versus human raters: mean difference = +0.93; proportional bias test *p* < 0.001.

Composite score agreement with human values was substantial and similar across the three LLMs (ICC for GPT‐5 = 0.69 [95% CI = 0.66 to 0.72]; Claude Sonnet 4 = 0.68 [95% CI = 0.60 to 0.74]; Gemini 2.5 Pro = 0.65 [95% CI = 0.35 to 0.79]; Figure 1). Bland–Altman plots for composite scores are presented in Figure 3A–C. The negative mean below 0.00 for GPT‐5 (Figure 3A) indicates this model (*μ*_GPT‐5_ = 11.65, SD = 2.02) returned modestly lower (*p* = 0.002; *η* ^2^ = 0.01, small effect) composite scores than human raters (*μ*_Human_ = 11.79, SD = 2.00). Claude Sonnet 4 (Figure 3B) and Gemini 2.5 Pro (Figure 3C) returned slightly higher (*p* < 0.001) composite scores (*μ*_Claude Sonnet 4_ = 12.32, SD = 2.02, *η* ^2^ = 0.12, medium effect; *μ*_Gemini 2.5 Pro_ = 12.72, SD = 1.75, *η* ^2^ = 0.31, large effect) than humans, overall. The statistical test for proportional bias was nonsignificant for GPT‐5 (*p* = 0.566) and Claude Sonnet 4 (*p* = 0.653).

The trend line for Gemini 2.5 Pro (Figure 3C) had a downward slope. The model assigned higher MERSQI scores than humans, mainly among studies with lower human MERSQI scores. This difference decreased as human MERSQI scores increased. The statistical test for proportional bias was significant for Gemini 2.5 Pro (*p* < 0.001), indicating substantial variation in agreement with human‐generated composite scores across the range of values.

## 4 Discussion

We evaluated the use of LLMs to appraise the quality of medical education research studies using the MERSQI tool. Among the three LLMs evaluated (GPT‐5, Claude Sonnet 4, and Gemini 2.5 Pro), no single model outperformed another across all six MERSQI domains. The highest score agreement was observed among all three LLMs and humans when evaluating the domains of study design and type of data. The LLMs were less accurate in appraising the domains of sampling, validity evidence, and data analysis. All three LLMs showed substantial and similar agreement with human composite scores. GPT‐5 tended to assign slightly lower scores than humans, whereas Claude Sonnet 4 and Gemini 2.5 Pro assigned higher composite scores. Notably, Gemini 2.5 Pro's scoring varied depending on study quality, whereby it rated lower‐quality studies more favorably and higher‐quality studies less favorably than humans, suggesting that Gemini 2.5 Pro may be more lenient when uncertain.

### 4.1 Comparisons With Prior Works

Within the broader context of using LLMs to support research and scholarly work, studies have demonstrated varying degrees of success, with some showing significant time savings and others highlighting the need for careful validation of AI‐driven decisions [28, 29, 30]. Consistent with our findings, RobotReviewer, a critical appraisal tool for classifying the risk of bias in clinical trials, has also demonstrated moderate to fair agreement with human raters (Cohen's *κ* ≤ 0.60), with large variability between domains [31, 32]. Likewise, evaluations of earlier ChatGPT models applying the Cochrane Risk of Bias 2.0 framework yielded weak results, with fair overall agreement (Cohen's *κ* = 0.24) and domain‐level agreement ranging from fair to slight [33, 34].

### 4.2 Practical Implications

Our findings suggest that current LLMs cannot independently perform accurate quality appraisals across all domains of interest and are best suited as assistive or pre‐screening aids under human supervision. Our collective findings also underscore the value in using domain‐level MERSQI scores over composite scores in isolation. The use of AI‐generated composite scores alone may mask the effects of compensatory scoring, whereby overinflated scores in one domain may cancel out underinflated scores in another domain. Hence, composite MERSQI scores from a given LLM may closely approximate human composite scores, despite discrepancies at the domain level.

It is probable that iterative prompt revisions could lead to improved appraisals. This is an area of ongoing investigation by our study team. For example, it may be helpful to revise the prompt to more explicitly address domains with low agreement. Such revisions could include examples and nonexamples, more detailed instructions or scoring rubrics, and chain‐of‐thought reasoning that decomposes tasks into sub‐tasks (e.g., to analyze validity using a separate prompt). A prompting protocol might also require the LLM to analyze the same text two or three times. While this approach would increase the overall cost, this investment may be justifiable if performance substantially improves.

While our findings suggest that fully automated independent appraisals of medical education research quality by current LLMs cannot fully replace human appraisals, there may be practical use cases that leverage human‐AI collaborations to maximize research efficiencies. For example, LLMs could serve as a second reviewer for domains where stronger alignment with human raters was observed (e.g., study design, type of data), thereby offsetting the time commitment required of a second human rater to review the domains where LLMs underperformed. While not ideal, this division of labor reduces the overall cost and time required to complete quality appraisals [35, 36, 37]. The value of such an arrangement is amplified for clinician‐educators whose protected research time is limited. In emergency medicine, where systematic reviews are commonly performed by small academic teams balancing clinical, educational, and scholarly responsibilities, a partial reduction in duplicate‐review burden could meaningfully expand what these teams are able to synthesize and how often [38].

### 4.3 Limitations

Several limitations should be acknowledged. The present findings are unlikely to generalize to other LLMs (e.g., Perplexity, Llama) or future GenAI models. Further investigation is needed to understand the mechanisms underlying the discrepancies between humans and LLMs, and to test whether fine‐tuning or targeted calibration of LLMs could improve domain‐level accuracy [39, 40].

Medical education research encompasses a diverse range of reporting standards and study designs, including qualitative studies, quasi‐experimental research, and curriculum evaluations, among others. Herein, studies were not classified by design type and, therefore, it is unknown whether LLM scoring varies across different study designs.

It is unclear whether LLMs' abilities to analyze multimodal inputs (e.g., figures, diagrams, tables) would have improved or diminished appraisal accuracy in this study. Visual elements often encapsulate details that are not fully conveyed in the text itself. As such, an LLM's incorrect interpretation of visual elements could systematically disadvantage studies that rely heavily on visual data [41, 42].

Although nearly all studies (97.0%; 1380/1423) included in this dataset were independently appraised by two human reviewers, variability in judgment or operational definitions may have influenced the scoring outcomes. Broader validation against larger expert panels or crowdsourced groups (e.g., > 1000 raters) would provide stronger benchmarks for evaluating human‐AI agreement [43, 44, 45, 46, 47]. Similarly, exploring intra‐model variability using a test–retest design is warranted.

### 4.4 Future Directions

Though the MERSQI tool is widely used in health professions education, the literature would benefit from investigating LLM outcomes using other appraisal tools (e.g., GRADE tool). Furthermore, given the MERSQI tool is best suited for quantitative research, future studies could extend this work by evaluating the application of LLMs to appraise qualitative research using tools such as Popay et al.'s framework [48]. GenAI's evolving ability to execute multimodal document parsing also warrants separate investigations of accuracy and reliability.

Lastly, the present study's design did not permit a critical investigation of diagnostic accuracy (i.e., sensitivity, specificity, and area under the ROC curve) related to rejecting or accepting manuscripts for publication based on their AI‐generated MERSQI scores. Investigating this information in future works could have implications for medical education journals exploring AI‐assisted screening mechanisms for managing manuscript quality control.

## 5 Conclusions

No single LLM (GPT‐5, Claude Sonnet 4, or Gemini 2.5 Pro) consistently outperformed the others across all six MERSQI domains. The LLMs showed moderate or fair agreement with human raters in three to four domains, indicating that human oversight is still essential for most aspects of quality appraisal. These findings encourage the medical education research community to thoughtfully consider the delicate balance between leveraging LLMs as research assistants and maintaining the essential role of human expertise.

## Author Contributions

**David A. Cook:** conceptualization, investigation, writing – original draft, data curation, methodology. **Adam B. Wilson:** conceptualization, writing – original draft, writing – review and editing, investigation, methodology, supervision, formal analysis, visualization. **Emily Rush:** conceptualization, investigation, writing – original draft, writing – review and editing. **Bernard Landry‐Wegener:** conceptualization, data curation, investigation, writing – review and editing. **John Swope:** software, data curation, writing – review and editing. **Mohammad Aldalou:** conceptualization, investigation, writing – original draft, methodology, writing – review and editing, data curation, resources. **William S. Brooks:** conceptualization, investigation, writing – original draft, writing – review and editing, project administration. **Corey Bills:** conceptualization, investigation, writing – review and editing, data curation.

## Funding

The authors have nothing to report.

## Conflicts of Interest

The authors declare no conflicts of interest.

## Supporting information


**Table S1:** Database.

**Data S1:** Methods supplement: prompt text.

**Data S2:** Supplementary index.

## Data Availability

The data that support the findings of this study are available from the corresponding author upon reasonable request.
