# Supplementary material for: Using Generative AI to Appraise the Quality of Medical Education Research Studies: Agreement Between AI‐Generated and Human MERSQI Scores
Source: AEM Educ Train. 2026 May 14;10(3):e70189. doi: 10.1002/aet2.70189 (PMC13176095; doi:10.1002/aet2.70189)
Supplement: Supplementary file 2 — Data S1: Methods supplement: prompt text. [file AET2-10-e70189-s003.pdf]

Methods Supplement: Prompt Text

```python
You are an expert researcher in the field of medical education and are asked to critically evaluate the quality of an empirical research study.

Please score the following academic article using the MERSQI (Medical Education Research Study Quality Instrument) rubric.

For each of the MERSQI rubric's six domains, assign the score that best fits the article’s methodology. In addition to providing domain scores, sum the six domain scores together to compute a single overall composite score. The maximum possible composite score is 18.

Only base the scores on what is explicitly stated or can be reasonably inferred from the text, tables, and/or figures.

Return your output strictly in JSON format with the following fields:

- "title": the title of the article
- "year": the year of publication
- "summary": a one-sentence summary of the article
- "authors": a list of authors
- "rubric_scores": an object containing each category and its score
- "total_score_ai": the total MERSQI score out of 18
- "justification": an object containing a brief explanation for each score

Here is a sample output:

{sample_output}

Here is the MERSQI rubric:
==================================================
{rubric_text}
==================================================
Here is the article:
==================================================
{article_text}
==================================================
```

Sample Output:

Python
```
{
  "title": "Improving Medical Education Through Simulation",
  "year": "2025",
  "summary": "This study evaluated the effectiveness of a new medical education program through simulation.",
  "authors": ["Jane Doe", "John Smith"],
  "rubric_scores": {
    "Study Design": 3,
    "Sampling: Institutions": 1.5,
    "Sampling: Response Rate": 1.5,
    "Type of Data": 3,
    "Validity Evidence": 2,
    "Data Analysis: Sophistication": 2,
    "Data Analysis: Appropriateness": 1,
    "Outcome": 2
  },
  "total_score": 16,
  "justification": {
    "Study Design": "Randomized controlled trial was used.",
    "Sampling: Institutions": "Participants were drawn from three institutions.",
    "Sampling: Response Rate": "Reported response rate was 78%.",
    "Type of Data": "Objective assessment data was used.",
    "Validity Evidence": "Reported internal consistency and relationships to other variables.",
    "Data Analysis: Sophistication": "Used regression analysis.",
    "Data Analysis: Appropriateness": "Statistical methods were appropriate for the design.",
    "Outcome": "Measured actual behavior changes in clinical settings."
    }
}
```

Rubric Text:

Python
```
MERSQI Scoring Rubric (Total Maximum Points: 18)
Study Design (max 3 points):
1 point: Single-group cross-sectional or posttest only
Definition: A single-group study conducted at one point in time or after the
```

intervention.

1.5 points: Single-group pretest and posttest
Definition: A single-group study with measurements before and after the intervention.

2 points: Nonrandomized, two-group
Definition: A study comparing two groups without random assignment (e.g., cohort or case-control study).

3 points: Randomized controlled trial
Definition: A study comparing groups with random assignment to intervention and control.

Sampling: Institutions (max 1.5 points):
0.5 points: 1 institution
Definition: Participants drawn from a single institution.

1 point: 2 institutions
Definition: Participants drawn from two institutions.

1.5 points: 3 or more institutions
Definition: Participants drawn from three or more institutions.

Sampling: Response Rate (max 1.5 points):
0.5 points: <50% or not reported
Definition: Less than 50% of eligible participants responded, or response rate not stated.

1 point: 50–74%
Definition: 50–74% of eligible participants responded.

1.5 points: ≥75%
Definition: 75% or more of eligible participants responded.

Type of Data (max 3 points):
1 point: Assessment by study participants
Definition: Data collected from participants' self-reports (e.g., surveys, interviews).

3 points: Objective data
Definition: Data collected objectively (e.g., observer ratings, standardized tests, performance measures).

Validity Evidence (cumulative points, up tomax 3 points):

+1 point: Content Validity
Definition: Evidence from theory, guidelines, experts, or existing instruments to justify the content of the measurement tool.

+1 point: Internal Structure
Definition: Evidence of reliability (e.g., internal consistency, interrater reliability) or factor analysis.

+1 point: Relationships to Other Variables
Definition: Evidence showing that the instrument correlates with other variables or distinguishes between expert and novice groups.

Data Analysis: Sophistication (max 2 points):
1 point: Descriptive analysis only
Definition: Analyses that summarize data (e.g., frequencies, means, medians) without inferential testing.

2 points: Beyond descriptive analysis
Definition: Analyses involving inferential statistics (e.g., regression, t-tests, ANOVA).

Data Analysis: Appropriateness (max 1 point):
0 point: Inappropriate or errors present
Definition: Statistical errors present or analysis inappropriate for the study design.

1 point: Appropriate
Definition: Analysis appropriate for the study design and type of data collected.

Outcome (max 3 points):
1 point: Satisfaction, attitudes, perceptions, opinions, general facts
Definition: Outcomes include participant satisfaction, attitudes, or basic facts.

1.5 points: Knowledge or skills

Definition: Outcomes focus on knowledge acquisition or skills (e.g., via tests or simulations).

2 points: Behaviors
Definition: Outcomes measure actual behaviors in real-world contexts.

3 points: Patient/healthcare outcomes
Definition: Outcomes measure effects on patient health, healthcare processes, or broader societal impact.
