# Supplementary material for: Using Generative AI to Appraise the Quality of Medical Education Research Studies: Agreement Between AI‐Generated and Human MERSQI Scores
Source: AEM Educ Train. 2026 May 14;10(3):e70189. doi: 10.1002/aet2.70189 (PMC13176095; doi:10.1002/aet2.70189)
Supplement: Supplementary file 3 — Data S2: Supplementary index. [file AET2-10-e70189-s001.pdf]

Using generative AI to appraise the quality of medical education research studies: Accuracy of automated MERSQI scoring

Supplementary_Index

- Methods_Supplement.pdf
  Prompt text and scoring rubric used to generate MERSQI ratings with large language models. (PDF, 5 PAGES, 149 KB).

- Database_S1.xlsx
  Dataset of studies and their MERSQI score sources used in this study. (Microsoft Excel worksheet, 180 KB).
